# Grossesse cervicale à 7 semaines d’aménorrhée: défis de la prise en charge

**DOI:** 10.11604/pamj.2017.26.3.11055

**Published:** 2017-01-04

**Authors:** Imane Khachani, Mohamed Hassan Alami, Rachid Bezad

**Affiliations:** 1 Maternité des Orangers, Université Mohammed V Rabat, Maroc

**Keywords:** Grossesse cervicale, césarienne, méthotrexate, hystérotomie, Cervical pregnancy, caesarean section, methotrexate, hysterotomy

## Abstract

La grossesse cervicale est une forme extrêmement rare de grossesse ectopique qui engage le pronostic vital maternel en raison du risque important d'hémorragie. Nous rapportons l'observation d’une patiente âgée de 35 ans, ayant accouché une première fois par césarienne et qui a présenté une grossesse cervicale diagnostiquée à 7 semaines d'aménorrhée. La prise en charge au sein de notre structure a fait appel au traitement médical puis chirurgical après échec du premier. L'intérêt de cette observation réside dans la démarche diagnostique et les différentes étapes de prise en charge thérapeutique.

Cervical pregnancy is an extremely rare form of ectopic pregnancy which can be life-threatening due to the high risk of hemorrhage. We report a case of a 35-year-old woman, who first gave birth by caesarean section, with cervical pregnancy diagnosed at 7 weeks of amenorrhea. Patient management within our structure was based on medical treatment followed by surgery after failure of medical treatment. The importance of this study lies in the diagnostic approach and in the different stages of therapeutic management.

## Introduction

L'incidence des grossesses ectopiques varie considérablement en fonction des populations étudiées [1]. La plupart sont de siège tubaire, et seules 5% concernent d'autres sites tels que l'ovaire, l'abdomen ou le col de l'utérus [2]. La grossesse cervicale constitue une forme extrêmement rare de grossesse ectopique et concerne en moyenne 1/20 000 grossesses [3, 4]. Décrite pour la première fois en 1817 par Sir Evrard Home, elle se définit par l'implantation du blastocyste au dessous de l'orifice interne du col [5]. Sa gravité est essentiellement liée au risque de complications hémorragiques, pouvant mettre en jeu le pronostic vital de la patiente. Nous rapportons ici un cas de grossesse cervicale révélé par métrorragies du premier trimestre, traité d'abord médicalement avec échec, puis par traitement chirurgical. Nous explorerons les facteurs de risque de cette pathologie, les modalités diagnostiques et les options thérapeutiques existantes.

## Patient et observation

**Figure 1:**
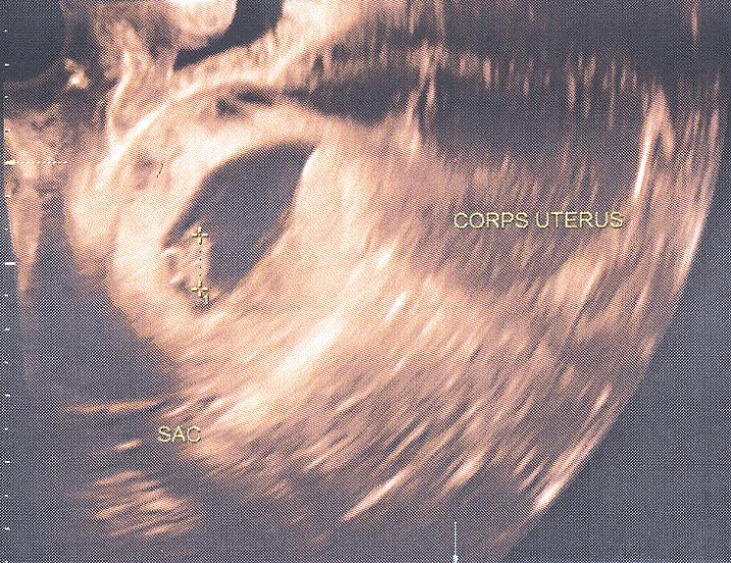
Image échographique transabdominale à 7SA+ 4 jours

Nous rapportons le cas d'une patiente âgée de 35 ans, au cycle régulier, II^ème^ geste, avec un enfant vivant accouché par césarienne programmée (césarienne de convenance, segmentaire basse). La seconde grossesse a été marquée par l'apparition à 7 semaines d'aménorrhée (SA) de métrorragies modérées, ayant motivé une consultation dans notre structure. L'examen clinique était sans particularités: la patiente était stable sur le plan hémodynamique, avec une tension artérielle à 120/70 mmHg, et un pouls à 78 pulsations/minute. L'examen de l'abdomen retrouvait une cicatrice de type Pfannenstiel; avec une palpation souple, sans douleur ni sensibilité localisées et un utérus de taille subnormale. L'échographie obstétricale par voie trans-abdominale a permis d'objectiver un utérus antéversé, légèrement augmenté de taille (mesurant 87x36mm), avec présence d'un sac gestationnel tonique, faisant 39mm de diamètre, siégeant au niveau du canal cervical, avec un pôle supérieur affleurant l'orifice interne du col. Le sac contenait un embryon avec activité cardiaque positive et longueur cranio-caudale de 10mm, correspondant à un âge gestationnel de 7SA+4 jours (Figure 1). Il n'y avait pas d'épanchement péritonéal. Le taux d'hCG plasmatique était de 134 404 mUi/mL.

Nous avons tenté un traitement médical en première intention par Méthotrexate, à raison d'1mg/kg en injection intramusculaire, répétée 48 heures plus tard, après réalisation d'un bilan sanguin (Numération formule sanguine, Taux de transaminases, Urée et Créatinine) qui était normal. Au troisième jour après l'administration de Méthotrexate, nous avons réalisé un contrôle échographique qui a objectivé la présence d'un sac gestationnel de 45mm de diamètre, siège d'un embryon avec activité cardiaque toujours positive, de longueur cranio-caudale projetant l'âge gestationnel à 8 SA+3 jours. La patiente était toujours stable, et avait présenté un seul épisode de métrorragie minime depuis le début de son hospitalisation. Nous avons alors décidé de réaliser un traitement chirurgical par laparotomie: après reprise de l'ancienne cicatrice de type Pfannenstiel et ouverture de la cavité, nous avons trouvé à l'exploration du pelvis une vessie ascensionnée sur la partie antérieure de l'utérus, avec un aspect normal de la cicatrice, sans signes d'invasion trophoblastique du myomètre. Les annexes étaient sans particularités. Nous avons réalisé dans un premier temps une ligature des artères hypogastriques, puis décollé progressivement la vessie de la face antérieure de l'utérus, jusqu'à libération complète du site de l'hystérorraphie. Nous l'avons incisée sur environ 3 centimètres, et procédé à l'aspiration de l'oeuf en l'abordant par son pôle supérieur. Nous avons ensuite soigneusement vérifié la vacuité utérine et assuré la libération complète des zones d'insertion du sac gestationnel. La mini-hystérotomie a été reprise par des points séparés et l'hémostase a été assurée sans difficultés. Nous avons fermé après libération des ligatures vasculaires avec des suites opératoires immédiates simples. La sortie de la patiente a été autorisée au 5ème jour post-opératoire, après une échographie de contrôle ayant objectivé un utérus de taille normale (74x31mm) avec endomètre fin et régulier, mesurant 6mm d'épaisseur et une cavité utérine libre. La surveillance de l'hCG plasmatique par la suite a objectivé une diminution progressive, jusqu'à négativation 7 semaines après l'intervention.

## Discussion

La grossesse cervicale est une grossesse ectopique rare. Des critères précis ont été élaborés par Rubin au siècle dernier pour affirmer son diagnostic, incluant [6]: la présence de glandes endocervicales en regard de l'insertion placentaire; la pénétration endocervicale des villosités choriales; la localisation de tout ou d'une partie du placenta sous le niveau de pénétration des vaisseaux utérins ou sous le niveau de réflexion du péritoine sur les faces antérieures et postérieures de l'utérus et l'absence de parties foetales à l'intérieur de la cavité corporéale. Elle constitue la plus rare des localisations ectopiques de grossesse et sa fréquence est très variable selon les séries publiées dans la littérature: entre 1/1000 et 1/95 000 grossesses, avec une fréquence moyenne de 1/20 000 grossesses [3, 4, 7]. Plusieurs facteurs de risque lui sont attribués, liés à une altération de la qualité de la muqueuse endométriale et à son incapacité à assurer la nidation de l'oeuf. Il s'agit notamment des antécédents de césariennes comme pour le cas de notre patiente, de curetages ou d'endométrite. Les anomalies structurales du col et du corps utérin, ainsi que le port de dispositif intra-utérin ont aussi été rapportés [7]. La grossesse par fécondation in vitro a également été associée à quelques cas rapportés dans la littérature [8]. Mais comme l'explique Singh, la rareté de cette pathologie, et l'existence de séries limitées ne permet d'identifier aucune cause directe, seulement des situations prédisposantes [9].

Les signes cliniques se limitent souvent à des métrorragies indolores et l'examen n'est pas d'une grande utilité pour le diagnostic: le col est parfois mou et bombé et l'utérus de taille subnormale à la palpation, sans douleur associée, ni sensibilité [7]. C'est l'échographie qui va permettre d'affirmer le diagnostic, en retrouvant les éléments suivants [10]: un sac gestationnel et trophoblaste entièrement situés sous l'orifice interne du col, repérés par le niveau de pénétration des artères utérines; Une cavité utérine vide; Un col dilaté “en tonneau” et une taille subnormale de l'utérus. L'échographie par voie endovaginale permet un diagnostic plus précoce, souvent problématique dans les grossesses à localisation ectopique comme le soulignent Parker et al. dans leur récente méta-analyse [11]. Cependant, selon Sherer et al. la voie transabdominale permet une meilleure visualisation de la localisation exacte du sac gestationnel et de ses rapports, afin de distinguer grossesse cervicale vraie et grossesse cervico-isthmique, ou grossesse sur cicatrice de césarienne, entités au pronostic différent [7, 12]. L'apport de l'échographie 3-D à contribué à l'amélioration du diagnostic, en offrant la possibilité de réalisation des coupes coronales, qui renseignent avec une meilleure précision sur le niveau d'implantation du sac gestationnel [13]. L’évolution naturelle de la grossesse cervicale se fait vers l'avortement et la rupture, sources d'hémorragie grave pouvant mettre en jeu le pronostic vital de la patiente, d'ou la nécessité d'une prise en charge rapide.

Il n'existe pas à ce jour de conduite à tenir codifiée pour la prise en charge de la grossesse cervicale. Le traitement classique reposait sur l’hystérectomie, avec des complications per-opératoires, une mortalité et des morbidités non négligeables [7]. La précocité du diagnostic, améliorée considérablement par l'échographie a permis le développement de traitements conservateurs, si bien qu'entre 1978 et 1994, dans l'une des plus grandes séries de grossesses cervicales, publiée par Ushakov et al., le taux d'hystérectomies retrouvé ne dépassait guerre les 22% pour les 120 cas colligés [14]. Les principales thérapeutiques rapportées dans les nombreux cas publiés par la suite sont: le traitement médical, à base de Méthotrexate (MTX), utilisé à la posologie de 1 mg/kg en injection intramusculaire et en association avec de l’acide folinique soit à visée curative seule, soit en préparation à une intervention endoscopique ou chirurgicale. Il peut également être utilisé en injection intraamniotique échoguidée [15]. Selon Hung et al. son efficacité est limitée par l'âge gestationnel supérieur à 9 SA, l'existence d'un embryon avec activité cardiaque positive, la longueur cranio-caudale supérieure ou égale à 10mm et le taux d'hCG plasmatique initial supérieur à 10 000 mUi/mL [16]. Sur ces 4 critères, 3 étaient retrouvés chez notre patiente, ce qui explique l'échec du traitement médical initié en première intention. Par ailleurs, ils rapportent que le foeticide, préalable ou concomittant au traitement par MTX permettrait un meilleur taux de succès [16]. Le curetage cervical suivi d’une méthode de compression, par sonde de Foley semble être la méthode la plus fréquemment décrite dans la littérature, avec des préparations ou associations variables, visant un meilleur taux de succès de l'intervention [9, 17]. Ainsi, Fylstra et al. et Iloki et al. y ont associé un cerclage préventif du col, afin d'améliorer la qualité de l'hémostase [17, 18]. En effet, le curetage cervical a lui seul expose à des risques majeurs d'hémorragie cataclysmique, vue la faible capacité de rétraction du col et donc d'hémostase spontannée. Hu et al. y ont associé une embolisation des artères utérines, entre 24 et 72 heures avant la réalisation du curetage, avec un taux de succès de 100% pour les 19 cas de leur série [19]. Ash et al. ont décrit en 1996 une prise en charge par résection hystéroscopique avec succès [20]. Cette technique a été reprise par plusieurs auteurs, soit en première intention, soit après échec du traitement médical. Un cas exceptionnel de traitement hystéroscopique sélectif a été rapporté par Jozwiak et al. sur grossesse gémellaire après fécondation in-vitro et implantation d'un embryon en intra-utérin et de l'autre au niveau cervical. La poursuite de la grossesse a été possible, jusqu'au terme, pour l'embryon intra-utérin [8]. Enfin, **l'hystérectomie** garde ses indications dans les diagnostics tardifs, ou dans les cas d'hémorragies non jugulables par d'autres moyens thérapeutiques. Par ailleurs, le traitement par laparotomie semble délaissé au profit de thérapeutiques moins invasives, mais celui-ci garde toujours ses indications, en fonction des moyens de prise en charge disponibles et des méthodes thérapeutiques les mieux maitrisées par l'équipe soignante, afin d'assurer à la patiente la meilleure sécurité thérapeutique possible. Peu de travaux explorent le pronostic en matière de fertilité ultérieure des patientes prise en charge pour grossesse cervicale. Bien qu'aucun cas de récidive n'ait été publié à ce jour, il convient cependant d'être prudent et d'aviser la patiente que les mêmes facteurs de risque associés à cette pathologie (antécédents de césarienne, curetage etc.) imposent une prise en charge précoce d'une grossesse ultérieure, pour un diagnostic rapide de la localisation de celle-ci.

## Conclusion

La grossesse cervicale est la plus rare des grossesses ectopiques et son diagnostic clinique est souvent difficile. Elle est pourvoyeuse de complications pouvant mettre en jeu le pronostic vital et de fertilité de la patiente. L'apport de l'échographie endovaginale et transabdominale a permis une prise en charge à des âges gestationnels plus précoces et un meilleur pronostic fonctionnel, avec la réalisation de moins en moins fréquente d'hystérectomies. Les thérapeutiques actuellement proposées combinent le plus souvent traitement médical à base de Méthotrexate et curetage ou résection hystéroscopique. Elle demeure un diagnostic certes rare mais à garder en tête devant des métrorragies du premier trimestre, en particulier chez les patientes à risque.
